# A questionnaire to measure the quality of midwifery care in the postpartum period from women’s point of view: development and psychometric testing of MMAYpostpartum

**DOI:** 10.1186/s12884-021-03857-8

**Published:** 2021-06-02

**Authors:** Mirjam Peters, Petra Kolip, Rainhild Schäfers

**Affiliations:** 1 Bielefeld School of Public Health, Bielefeld University, Bielefeld, Germany; 2 Department of Applied Sciences, The Hochschule für Gesundheit, University of Applied Sciences (hsg), Bochum, Germany

**Keywords:** Midwifery care, Quality of care, Postpartum, Patient perspective, Psychometrics, Patient satisfaction, Evaluation of care, Respectful care, Midwifery home care, Postnatal

## Abstract

**Background:**

Home postpartum care is a major part of midwifery care in Germany. The user perspective plays an increasingly important role in the evaluation of health services, but there is a lack of valid and theoretically based measuring instruments, especially in midwifery care. The aim of this study was to develop and validate an instrument for measuring quality of midwifery care in the postpartum period from the perspective of women.

**Methods:**

The following steps were taken to achieve this: (1) definition of the goals of midwifery work; (2) literature-based item development; (3) item selection based on a pre-test (*n* = 16); (4) item reduction and investigation of factor structure by means of explorative factor analysis (EFA; *n* = 133);(5) second EFA (*n* = 741) and confirmatory factor analysis (CFA; *n* = 744) based on a split representative sample survey; (6) hypothesis-based testing of correlations to sociodemographic characteristics of women and to characteristics of care.

**Results:**

Measurement of Midwifery quality postpartum (MMAYpostpartum) consists of three scales with a total of 17 items which were found to have acceptable internal consistency: Personal Control (Cronbach’s alpha = .80), Trusting Relationship (Cronbach’s alpha = .87) and Orientation and Security (Cronbach’s alpha = .78). CFA verified and confirmed three factors: CFI = .928, TLI = .914, RMSEA = 0.073.

**Conclusion:**

MMAYpostpartum is a predominantly valid, reliable short tool for evaluating the quality of midwifery care postpartum. It can be used to evaluate midwifery care, to compare different care models and in intervention research. It thus supports the orientation of midwives’ work towards the needs of women and their families.

## Background

In Germany, midwives support women from the beginning of pregnancy until the end of breastfeeding. During pregnancy, they have a secondary role compared to obstetricians. During childbirth, a midwife must be present by law. In most cases, gynecologists are also present and delivery rooms are supervised by gynecologists. Most women go home 3 to 5 days after giving birth. Postpartum home care is exclusively offered by midwives.

Women can receive up to 36 home visits postnatally and during the breastfeeding period [1]. In Germany, 92.8% of women use home-based care provided by a midwife in the postpartum period, with lower use in vulnerable groups.

It typically involves an average of 12 visits (SD ± 5.7), of 38.8 min duration (SD ± 17.4) and continues for up to 9 weeks after birth (SD ± 3.7) or until the end of the breastfeeding period [2]. Care during this period is offered by freelance midwives. It is currently not subjected to formal evaluation (or quality control).

The user’s perspective plays an increasingly important role in the evaluation of health services [3]. Evaluation from the user’s perspective appears to be particularly important in the area of home postnatal care. There is an explicit public health expectation in Germany that women and their families receive postpartum care. It is intended to support breastfeeding and help at risk women and families, as this phase of life is particularly important for the later health of the child [4]. Allowing home visits necessitates a special relationship of trust between women, families and the midwife, however.

Several instruments involving the user perspective to measure the level of satisfaction or the experience of midwifery care have already been developed [5–10]. A number of problems have been identified with these tools, however. For example, they are not very sensitive [11, 12], and satisfaction depends on the expectations of the user and tends to be measured on an emotional-affective level, and midwifery care evaluated from a “consumer experience” perspective [11, 13].

In contrast, very few instruments have been developed to measure the *quality* of midwifery care from a users’ perspective. In the field of midwifery, there is one instrument for measuring quality in antenatal care (QPCQ [11];, and one for measuring quality during pregnancy and childbirth (PCQ) [14]. No instrument for measuring quality in postpartum care was found in a scoping review [15].

Quality is understood here as the extent to which objectives are achieved in various defined dimensions of a health service [16, 17]. The dimensions of midwifery care are understood comprehensively. Topics such as disrespect and abuse/respectful care are just as important as health and medical topics or to give orientation for women and families in a potentially challenging life situation.

The measurement of quality can be used by professionals to evaluate, develop and professionalize their own work. It can also be used to compare different models of care or for evaluation within intervention research. In addition, midwives in Germany are required to evaluate their work, however no validated instrument is available yet [18].

A theory-based, valid and reliable assessment tool for quality in postpartum midwifery care from the point of view of women as users is needed.

## Method

The aim of this study is to develop and validate an instrument for measuring quality of midwifery care in the postpartum period from the perspective of women. For this purpose, the usual steps for questionnaire development and validation were followed.

The validation of the questionnaire took place as part of the study HebAB.NRW - Midwifery Care in North Rhine-Westphalia. The study was funded by the Landeszentrum für Gesundheit, NRW (LZG.NRW; funding code: LZG TG 72001/2016) [2]. The Ethics Committee of the University of Applied Sciences, Hochschule für Gesundheit in Bochum, approved the study. The authors are midwifery scientists and health scientists. Two of the authors have worked as midwives in the past.

An instrument for quality assessment during birth has also been developed and is currently under review.

### Phase one: Theoretical foundation

In this work quality was defined as the extent to which objectives are achieved. In order to be able to measure this, the objectives of midwifery were first defined. To this end, a systematic literature search was carried out on the objectives and concepts of midwifery and on the needs and wishes of women regarding midwifery care. The Walker and Avant method of *theory construction* [19] was used to develop a theory on the aims and purpose of midwifery. The procedure and results have been published in detail elsewhere [20].

### Phase two: item generation and selection

Items were developed for each of the midwifery goals defined in phase one. The wording of the items was guided by the literature on women’s needs for midwifery care. The literature from Phase 1 was used for this purpose.

A pre-test of the item list was performed with nine new mothers, five midwifery scientists and two midwives. The items were evaluated for clarity, relevance, acceptability, importance and freedom of overlap. Where appropriate, items were reformulated or removed.

Items were scored on a five-point Likert scale with a neutral centre in order to avoid systematic distortion by undecided or neutral participants. Due to the potential for ambiguous research results, a “not sure” category was omitted. The possible answers were as evenly distributed as possible and were as follows: “not applicable at all”, “not applicable”, “neither”, “applicable” and “fully applicable” [21].

### Phase three: item reduction and investigation of factor structure

#### Sampling

Since there were no prior assumptions about the data structure on which to base a power analysis and due to the small number of expected factors, a minimum sample size of 100 women was aimed for [22].

For this purpose, a convenience sample was used, with recruitment via a freely accessible link on social media. This allowed uncomplicated and low-threshold access to a diverse sample. Included were women over 18 years of age who had given birth to a child in the last 12 months, had taken advantage of postnatal midwifery care and given informed consent to participate. The items were presented in a randomized order to exclude sequence effects. The survey was conducted online with the software Unipark.

#### Data analysis: exploratory factor analysis (EFA)

Exploratory factor analysis (EFA) was used to reduce the number of items. A principal axis factoring analysis with Varimax rotation was performed using SPSS 24.0. The suitability of the data for a factor analysis was checked for using the Kaiser-Meyer-Olkin criterion (KMO > .5) and the significance of the Bartlett test of sphericity. Factors with an eigenvalue greater than 1 were selected.

Items whose level of difficulty was judged too low (<.20) or too high (>.80) were viewed critically, as were items which did not sufficiently load on the primary factor (< .50), or whose crossloadings were too high (> .45). In addition, attention was paid not only to the statistical parameters, but also to how well the item’s content fit the factors during selection. The internal consistency of the scales was evaluated using Cronbach’s Alpha (α), with an internal consistency of α < .75 being considered unacceptable.

The factor structure of the reduced number of items was examined using the principal axis factoring analysis with Varimax rotation.

### Phase four: questionnaire validation and determination of psychometric properties

#### Sampling

Phase four was conducted as part of a comprehensive study of the accessibility of midwifery care for women in North Rhine-Westphalia which took place between 01.02.2018 and 15.06.2018. In a retrospective cohort study, 45 of the 146 obstetric departments in North Rhine-Westphalia (NRW) were randomly selected. Twenty-seven clinics gave their consent to recruitment. North Rhine-Westphalia is the most populous state in Germany (17.9 mill. Inhabitants; ranked 7th in GDP per capita; ranked 11th in unemployment rate in Germany) [23].

Participants were asked about utilization, access, continuity and availability of midwifery care, as well as empowerment, self-rated health and use of e-health. The survey took place four to 12 months after birth. This period was chosen in order to generate a sufficiently large sample. The survey was started only 4 months after childbirth, so that an retrospective assessment of the quality of care in the postpartum and breastfeeding period was possible.

The women could participate online or by mail. It was also possible to participate online via open access for women who fulfilled the inclusion criteria but gave birth in a non-participating hospital. This opportunity was offered because the recruitment rates were lower than expected and there was also public interest in participation. Inclusion criteria were the provision of informed consent, having given birth in NRW during the survey period, age over 18 years and the use of midwifery postpartum care. The online survey was conducted with Unipark software.

#### Data analysis: confirmatory factor analysis (CFA)

Based on the existing data it was assumed that the implementation of the first EFA was not sufficiently stable due to the small sample size. Therefore the second large sample was divided into two subsamples to perform a cross-validation [24, 25]. An EFA was carried out on the first sub-sample andhe CFA on the second subsample.

Questionnaires with more than two missing items per scale were removed. In those missing one item it was replaced by the scale mean.

The final scale was then tested using Confirmatory Factor Analysis (CFA) using R 3.5.3. to test the relationship between the manifest variables and the underlying latent construct. The following quality criteria were used to ensure the construct validity (range in literature):

Comparative Fit Index (CFI > .90; .97), Tucker-Lewis Index (TLI > .90), goodness of fit index (GFI > .90; .95), adjusted goodness of fit index (AGFI > .85; .90), Standardized Root Mean Square Residual (SRMR <..10; .05), Root Mean Square Error of Approximation (RMSEA <.08; .05), Chi^2^/df < 3,0; 2,0, Factor Reliability (FR > 0.6), Average Variance Extracted (AVE > 0.5) [26, 27].

The internal consistency was determined using Cronbach’s alpha for the subscales and the overall scale, as well as Cronbach’s alpha if item deleted from subscale. There was no way to determine the criterion validity, as there is no gold standard for measuring quality of midwifery care in the postpartum period from the perspective of women.

### Phase five: construct/convergent validity

Whether there are differences between specific characteristics of women and maternity care and the MMAYpostpartum score was examined. For this purpose, the cases were dichotomized into those below the 25th and those above the 75th percentile of the MMAYpostpartum score.

It was hypothesized that the MMAY score would not correlate to the personal characteristics of the woman or child or with the number or duration of postpartum visits. In contrast, it was hypothesized that the MMAY score would correlate positively with satisfaction regarding the number of visits. Furthermore, the women were classified according to whether they had made negative comments on postpartum care in a free text section of the questionnaire. Negative comments were expected to correlate negatively with the MMAY score.

The entire sample of the second survey was therefore used. Variables of interest were dichotomized. Odds ratios were calculated and Pearsons Chi^2^ was used to test the significance of the correlations.

The following variables were included in the analysis. Characteristics of the woman/child: Urban/rural resident, born in Germany, German native speaker, vocational training, university entrance qualification, annual income above 2.500 € (~ 2.718 US$), multipara, spontaneous vaginal birth, twins, premature birth, breastfeeding, self-rated health (SF-1 [28]), self-rated health of child, self-rated mental health postpartum, self-rated physical health postpartum.

Characteristics of care: place of birth, antenatal care by a midwife, satisfaction with the number of postpartum visits, number of postpartum visits, duration of care in weeks, and private health insurance.

## Results

The results of the six phases are presented below.

### Phase one: development of a theoretical foundation

**Fig. 1:**
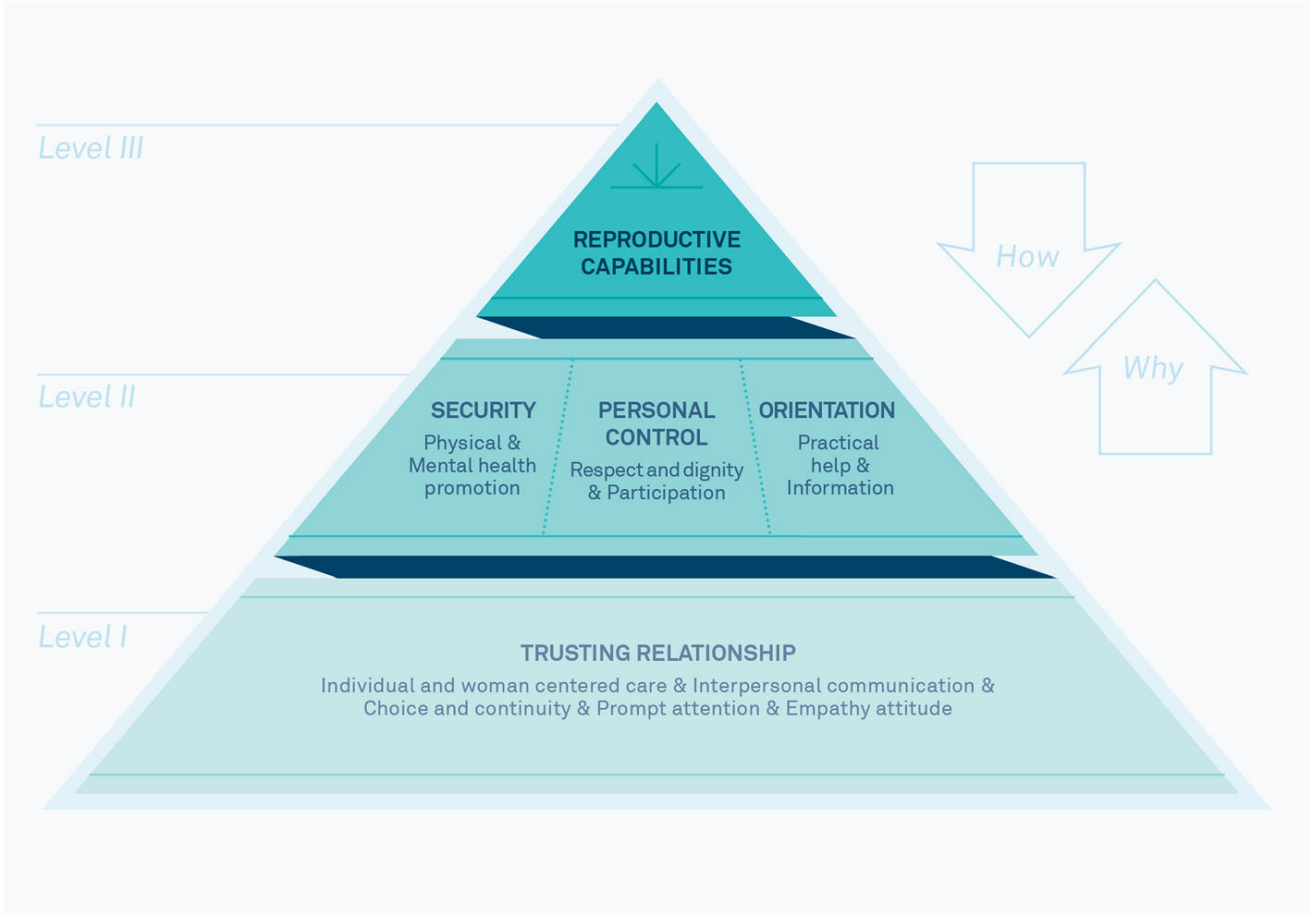
Hierarchical model of the means and targets of midwifery from [20] [Picture quote]

The theory of the goals and purpose of midwifery work is described in a three-level model. On the first level, the aim is to establish a *Trusting relationship*. This promotes the three goals on the second level: *Security, Personal control* and *Orientation*. These serve the purpose of midwifery on the third level, the *Promotion of the reproductive capabilities* of women and families. The theory thus shows a total of four goals of midwifery care (see Fig. 1).

### Phase two: item generation and selection based on a pre-test

On the basis of phase one, 145 items were developed. These were then revised based on comments made in the pre-test and reduced to 90.

### Phase three: item reduction and investigation of factor structure

#### Sample I

One hundred thirty-three women met inclusion criteria and took part in the online random sample (convenience sample). Sociodemographic and anamnestic data are shown in Table 1. It is noticeable that there is a small proportion of women with a migrant background and a small proportion of first-time mothers. The proportions of twin births and premature births do not reflect those in statistical routine data.

**Table 1:** Sociodemographic and obstetric characteristics of participants in phases three to five

	Sample I		Sample II						
	(*N* = 133)		Total (*N* = 1485)		EFA (*N* = 741)		CFA (*N* = 744)		*p*-value*
	n	%	n	%	n	%	n	%	
Mean age, years (SD)	29	(5,6)	33	(4,3)	33	(4,3)	33	(4,4)	0,2
**Migrant status**									
Born in Germany	127	(95,5)	1320	(88,9)	666	(89,9)	654	(87,9)	0,2
Not born in Germany	6	(4,5)	165	(11,1)	75	(10,1)	90	(12,1)	
Mother tongue: German	124	(93,2)	1334	(90,2)	671	(90,8)	663	(89,6)	0,4
Other	9	(6,8)	145	(9,8)	68	(9,2)	77	(10,4)	
**University entrance qualification**									
Yes	86	(65,2)	1193	(80,3)	596	(81,4)	597	(80,2)	0,9
No	46	(34,8)	274	(18,5)	136	(18,6)	138	(18,8)	
**Professional qualification**									
Yes	126	(94,7)	1446	(97,8)	722	(98,0)	724	(97,6)	0,6
No	7	(5,3)	33	(2,2)	15	(2,0)	18	(2,4)	
**Net monthly household income**									
< 2500 €	–		550	(38,1)	267	(37,1)	283	(39,0)	0,5
> 2500 €	–		894	(61,9)	452	(62,9)	442	(61,0)	
**Health insurance type**									
Public	104	(78,2)	1048	(71,1)	517	(70,2)	531	(71,9)	0,1
Private	11	(8,3)	194	(13,2)	104	(14,1)	90	(12,2)	
Additional private	18	(13,5)	233	(15,8)	115	(15,6)	118	(16,0)	
**Parity**									
Primipara	24	(18,6)	800	(45,2)	394	(54,3)	406	(44,7)	0,7
Multipara	105	(81,4)	659	(54,8)	331	(45,7)	328	(55,3)	
**Birth mode**									
Spontaneous	84	(67,7)	904	(61,8)	463	(63,2)	441	(60,3)	0,2
Forceps/vacuum	9	(7,3)	138	(9,4)	78	(10,6)	60	(8,2)	
Caesarean section	31	(25,0)	422	(28,8)	192	(26,2)	230	(31,5)	
**Twins/ Multiples**									
Yes	1	(0,8)	29	(98,0)	16	(2,2)	13	(98,2)	0,6
No	132	(99,2)	1449	(2,0)	722	(97,8)	727	(1,8)	
**Premature birth (< 37 SSW)**									
Yes	15	(11,3)	104	(7,0)	48	(6,5)	56	(7,6)	0,4
No	118	(88,7)	1377	(93,0)	692	(93,5)	685	(92,4)	
**Breastfeeding**									
Yes	126	(95,5)	1352	(91,4)	679	(91,6)	673	(91,1)	0,9
No	6	(4,5)	128	(8,6)	62	(8,4)	66	(8,9)	
**Self-rated health (SF-1)**									
Poor	–		22	(1,5)	12	(1,6)	10	(1,3)	1,0
Fair	–		125	(8,4)	65	(8,8)	60	(8,1)	
Good	–		551	(37,2)	275	(37,3)	276	(37,1)	
Very good	–		511	(34,5)	250	(33,9)	261	(35,1)	
Excellent	–		272	(18,4)	136	(18,4)	136	(18,3)	

*p-values based on chi-square und t-test comparing the two subsamples

#### Data analysis: EFA

The results of the KMO criterion (0.91) and the Bartlett test (*p* < 0.00) were considered suitable for conducting the initial EFA for item reduction. There were 12 factors with an eigenvalue > 1. This solution explains a variance of 78.1%, with the first factor explaining 56.79% of the total variance. Items were reduced from 90 to 17 as described above.

A further EFA was carried out to investigate the factor structure. The EFA of the remaining 17 items showed a KMO criterion of 0.93 and a significant Bartlett test (*p* < 0.00). Three factors with an eigenvalue > 1 yielded a variance explanation of 65.63%, with the first factor explaining 50.97% of the total variance. The factor loadings are shown in Table 2.

**Table 2:** Key indicators of the explorative factor analysis

Items		EFA I (*N* = 133)			EFA II (*N* = 741)		
		FPC	FTR	FOS	FTR	FOS	FPC
The midwife was friendly to significant others	1	.80	.25	.27	.74	.27	.12
Information was neutral/judgement free	2	.80	.18	.02	.64	−.01	.04
Lifestyle choices were respected	3	.80	.26	.22	.76	.17	.08
I felt judged	4	.73	.30	.20	.17	.06	.89
Privacy was respected	5	.71	.30	.40	.77	.17	.14
The midwife was organised	6	.22	.79	.22	.66	.31	.07
I received the right information at the right time	7	.33	.75	.29	.67	.39	.09
Examinations were performed without consent	8	.20	.73	.01	.05	.02	.90
The midwife took time to listen	9	.25	.73	.35	.69	.39	.15
I couldn’t speak freely about my feelings/fears	10	.26	.67	.21	.14	.21	.76
The midwife enabled me to connect with other women and families	11	.00	.09	.75	−.11	.52	.07
The midwife helped with physical complaints	12	.41	.43	.65	.37	.67	.06
The midwife helped me understand what was happening to my body	13	.39	.46	.63	.39	.64	.05
The midwife helped my partner adjust to his/her new role	14	.51	.28	.59	.17	.73	.04
The midwife respected my religion/ culture	15	.21	.07	.55	.20	.39	.06
I was able to choose a midwife	16	.15	.41	.53	.16	.40	.03
The midwife helped me deal with strong emotions	17	.48	.39	.46	.28	.72	.11
Eigenvalues		4.17	3.86	3.13	4.02	3.06	2.29
Variance%		24.5	22.7	18.4	23.7	18.0	13.4
Cumulative Variance%		24.5	47.2	65.6	23.7	41.7	55.1

This solution primarily shows the theoretically postulated factors. In the factor analysis it was not possible to distinguish between the two theoretically separate factors *safety* and *orientation*. The factors *Personal Control* (FPC) and *Trusting Relationship* (FTR) contain five items each and demonstrate good internal consistency (FPC: α. =.89; FTR: α. =.86). The factor *Security and Orientation* (FSO) contains seven items and displays good internal consistency of α. =.84. The internal consistency of the entire scale was rated very good (α. = .93).

### Phase four: questionnaire validation and determination of psychometric properties

#### Sampling

Three thousand one hundred one women gave their consent to participate in the HebAB.NRW study between 01.02.2018 and 15.07.2018. One thousand eight hundred seventy-three women completed the questionnaire, 1649 received postpartum care and 1485 (79.25%) could be included in the analysis of the scale.

The sample contains a relatively high proportion of women with high socioeconomic status (income, education) and with no migrant background. The two sub-samples do not differ significantly in sociodemographic and obstetric characteristics [Table 1].

#### Data analysis: EFA

The data (*n* = 741) was deemed suitable for EFA, based on the KMO criterion of 0.90 and a significant Bartlett-Test (*p* < 0.00). Three factors with an eigenvalue > 1 were found, showing a variance explanation of 55.09%; the first factor has a variance explanation of 23.65%. The results are shown in Table 2.

On the basis of the EFA and content aspects, the allocation of items to the scales *Personal Control* and *Trusting Relationship* was revised. Items 15 and 16 had very low loadings on all factors. Item 15 asked whether the midwife took into account the culture or religion of the woman. This item was retained because of its importance in terms of content. Item 16 asked whether the woman was able to choose a midwife. It was removed as a factor because of the loads and the difficulty of fitting the content to a factor.

#### Data analysis: CFA

The 16 item model and the revised factor structure were verified using a CFA with a sample of *n* = 744. The CFA confirmed an adequate model of fit. The fit indices for the final model were CFI = 0.928, TLI = 0.914, GFI = 0.94, AGFI = 0.91, RMSEA = 0.073, SRMR = 0.053, Chi^2^/df = 4.951, FR = 0.82 to 1.39 and AVE = 0.33 to 0.40. The model fit is therefore above the limit of < 2.5 and the AVE is too low. All other quality criteria are fulfilled.

### Phase five: construct/convergent validity

The data were examined for correlations between extreme values of the MMAYpostpartum score (<25th; >75th percentile) and characteristics of women/children and maternity care (*n* = 704; Table 3).

**Table 3:** Group comparison variables

	Chacteristic applicable		Chacteristic not applicable					
	n	%	n	%	*p*-value	Odds Ratio	lower	upper
**Sociodemographic characteristics**								
Rural resident	123/252	48.8	114/230	49.6	.87	0.97	0.68	1.39
Born in Germany	318/633	50.2	29/71	40.8	.13	1.46	0.89	2.41
German native speaker	320/634	50.5	16/69	37.7	.04*	1.69	1.01	2.81
Professional qualification	337/689	48.9	10/15	66.7	.17	0.48	0.16	1.42
University entrance qualification	289/568	50.9	57/129	44.2	.17	1.31	0.89	1.92
Annual income > 2500 €	206/420	49	136/268	50.7	.66	0.93	0.69	1.27
**Obstetric characteristics**								
Multipara	141/299	47.2	202/391	51.7	.24	0.84	0.62	1.13
Spontaneous vaginal birth	213/418	51	134/286	46.9	.29	0.85	0.63	1.15
Singleton	343/692	49.6	3/9^a^	33.3	.33	1.20	0.49	7.92
Term birth	335/658	49.1	23/44	52.3	.68	0.88	0.48	1.62
Breastfeeding	254/511	49.7	93/190	48.9	.86	1.03	0.74	1.44
SF1^b^	310/631	49.1	37/71	52.1	.63	0.89	0.54	1.50
Child’s health (at least good)	319/649	49.2	28/55	50.9	.80	1.07	0.62	1.86
Physical health postpartum (at least good)	157/312	50.3	189/390	48.5	.62	1.08	0.80	1.45
Mental health postpartum (at least good)	199/397	50.1	144/301	47.8	.55	1.10	0.81	1.48
**Characteristics of care**								
Hospital birth	330/662	49.8	17/42	40.5	.24	0.68	0.36	1.29
High number of postpartum visits^c^	141/302	46.7	206/402	51.2	.23	0.83	0.62	1.12
Long duration of care in weeks^c^	91/197	46.2	256/507	50.5	.31	0.84	0.61	1.17
Statutory health insurance.	244/486	50.2	103/2018	47.2	.47	0.89	0.65	1.22
Satisfaction with the number of postpartum visits	292/607	48.1	54/91	59.3	.046*	0.64	0.41	0.99
Negative annotations in free text fields	7/11	63.6	340/693	49.1	.34	0.55	0.16	1.90

* *p* < .05; ^a^Cell population too small to perform a valid X^2^-test,^b^ Self rates Health (SF1): excellent or very good; ^c^ above average

Women who were satisfied with the number of postpartum visits were significantly less likely to rate the care as poor. Women who had made negative comments in free text fields were more likely to rate the midwifery care as poor. This difference was not significant, but also had low cell occupancy.

As hypothesized, for most variables no significant correlations were found. Contrary to the assumptions made, there was a correlation between mother tongue (significant) and country of birth (not significant) and the assessment of the quality of care in the postpartum period. Women of a migrant background were less likely to rate the quality as poor. Contradictory and non-significant tendencies were found with regard to university entrance qualification and professional training and the assessment of quality. There was also a non-significant tendency for women who had given birth in a hospital to report poorer quality than women who had not.

### Characteristics of final scale

Measurement of Midwifery quality – MMAYpostpartum measures the quality of midwifery care with 16 items in three scales. Trusting Relationship measures whether the midwife is empathetic and respects the individual situation of the woman so that a trusting relationship can develop. This also includes good communication and organizational aspects. Orientation and Security measures the practical assistance and information provided in a potentially new and challenging life situation. In addition, it measures whether the midwife provides security by protecting and promoting the mental and physical health of the woman and her child. The subscale Personal Control measures the involvement of women in decision-making and the feeling that their own sovereignty and integrity are respected.

The scales and their properties are shown in Table 4. The scale Trusting Relationship is the only scale in which the possible range is not completely filled by the observed data. The internal consistency of the subscales and the total scale, with Cronbach’s Alpha ranging from α. =.78 to α. =.87, is acceptable to good. The properties of the individual items are described in Table 5. Two items were kept for content reasons, although deleting the item from the subscale would have improved Cronbach’s alpha.

**Table 4:** Characteristics and distribution of the 16-item MMAYpostpartum scale

Scale	Number of Items	Range (possible)	Range (observed)	Mean (SD)	Median	Cronbach’s Alpha
Trusting Relationship	7	7–35	9–35	31,28 (4,06)	32	.87
Orientation and Secruity	6	6–30	6–30	22,59 (4,11)	23	.78
Personal Control	3	3–15	3–15	12,54 (3,26)	14	.80
MMAY Score	16	16–80	25–80	65,95 (9,23)	68	.87

**Table 5:** Scale descriptions and psychometric properties

Scale and Item	Item num-ber	Mean	SD	Item-Total correlation coefficient	Scale internal consistency Cronbach’s Alpha	Cronbach’s Alpha if item deleted from subscale
**Factor Personal Control**						
I couldn’t speak freely about my feelings/fears	10	3.89	(1.37)	.53	.80	.86
Examinations were performed without consent	8	4.41	(1.20)	.69		.69
I felt judged	4	4.42	(1.25)	.74		.62
**Factor Trusting Relationship**						
The midwife was friendly to significant others	1	4.73	(0.56)	.72	.87	.85
Information was neutral/judgement free	2	4.25	(0.95)	.55		.87
Lifestyle choices were respected	3	4.59	(0.68)	.66		.85
Privacy was respected	5	4.62	(0.65)	.64		.86
The midwife was organised	6	4.29	(0.89)	.67		.85
I received the right information at the right time	7	4.27	(0.89)	.71		.85
The midwife took time to listen	9	4.52	(0.77)	.71		.85
**Factor Orientation and Secruity**						
The midwife helped with physical complaints	12	4.39	(0.81)	.65	.78	.72
The midwife helped me deal with strong emotions	17	3.87	(1.02)	.61		.72
The midwife helped me understand what was happening to my body	13	4.25	(0.89)	.69		.71
The midwife helped my partner adjust to his/her new role	14	3.67	(1.13)	.63		.71
The midwife enabled me to connect with other women and families	11	2.52	(1.23)	.29		.81
The midwife respected my religion/ culture	15	3.88	(0.98)	.38		.78

## Discussion

This study aimed to develop the first reliable and valid instrument for the assessment of the quality of home midwifery care postpartum. The MMAYpostpartum contains 16 items in three scales: Trusting Relationship, Orientation and Security, and Personal Control.

Overall, MMAYpostpartum demonstrates good reliability and validity with small weaknesses.

The content validity is estimated to be very high. Thus, a literature-based theory on the aims and purpose of midwifery work was carefully prepared especially for the development of the quality scale. The scales and items were then developed on the basis of this theory. In addition, the items and scales were evaluated by midwives and midwifery scientists before the pre-test. The face validity is supported by the item evaluation, which was carried out by mothers in the pre-test.

Exploratory and confirmatory factor analysis was performed to investigate construct validity and the internal relationship of the data. Differences in the assignment of items to the factors *Trusting Relationship* and *Personal Control* were found between the first and the second sample. The first sample was not representative and had a rather small number of participants, so it is thought that this could be responsible for the differences. The factor structure should therefore be investigated in further studies. The low values in AVE and slightly too high values in model fit could represent variances in the empirical data that are unexplained by the model. It is noted that these values were worse [6] or were not reported [11, 12, 14] in previous instruments developed to measure satisfaction, experience or quality from women’s points of view.

Two theoretically postulated factors (Orientation and Security) could not be separated empirically. This may indicate that this is a professional distinction made by midwives in their work, but that women do not distinguish between these two factors.

However, the data analyses clearly support the existence of three factors in overwhelming agreement with the theory, and the usual quality criteria, such as CFI, TLI and RMSEA, turned out well and support the construct validity of MMAYpostpartum.

Criterion validity was examined on the basis of postulated correlations to other items of the HebAB.NRW study, which were collected in connection with the MMAYpostpartum Score. The hypotheses were largely confirmed, so that the MMAY postpartum score predominantly shows no correlation with personal characteristics or with characteristics of care. Instead, it measures a construct of its own. As hypotheses, there is a logical connection between the assessment of quality of care and satisfaction with the quantity of care. However, a non-postulated connection between characteristics of a migrant background and the evaluation of quality was found. This should be investigated further. A non-significant trend was shown by the fact that women who had given birth in hospital more often rated the quality of postpartum care as poor than women who had given birth outside the hospital. This could be due to the fact that women in Germany who do not give birth in hospital are more likely to receive continuity of care or carer than women who give birth in hospital. In some cases, the cell populations were too small to make valid statements.

Good reliability in terms of internal consistency is supported by good Cronbach Alpha values.

Our study also has limitations. The questions are adapted to the German health system and were evaluated in Germany. The sample was not fully representative in sociodemographic terms. In addition, it was not possible to test the correlations with existing instruments. And no test-retest measurement to support reliability over time was possible due to the study design. The main strengths are to be found in the careful theoretical work done in advance, and in the rigorous execution of the statistical analyses.

## Conclusion

The 16 item MMAYpostpartum questionnaire is a predominantly valid, reliable short tool for evaluating the quality of midwifery care postpartum. It can be used to evaluate the work of midwives (or midwifery teams), to compare different care models and in intervention research. It thus supports the orientation of midwives’ work towards the needs of women and their families.

## Data Availability

Raw data of the Scala MMAYpostpartum can be requested from MP.

## Abbreviations

AGFI: Adjusted Goodness of Fit Index
AVE: Average Variance Extracted
CFA: Confirmative Factor Analysis
CFI: Comparative Fit Index
df: Degrees of freedom
EFA: Explorative Factor Analysis
FR: Factor Reliability
FPC: Factor Personal Control
FTR: Factor Trusting Relationship
FOS: Factor Orientation and Security
GFI: Goodness of Fit Index
KMO: Kaiser-Meyer-Olkin
LZG.NRW: Landeszentrum für Gesundheit, NRW
NRW: North Rhine-Westphalia
RMSEA: Root Mean Square Error of Approximation
SRMR: Standardized Root Mean Square Residual
SD: Standard deviation
